# Exploring structure-activity relationship of S-substituted 2-mercaptoquinazolin-4(3H)-one including 4-ethylbenzenesulfonamides as human carbonic anhydrase inhibitors

**DOI:** 10.1080/14756366.2020.1722121

**Published:** 2020-02-02

**Authors:** Adel S. El-Azab, Alaa A.-M. Abdel-Aziz, Hany E. A. Ahmed, Sivia Bua, Alessio Nocentini, Nawaf A. AlSaif, Ahmad J. Obaidullah, Mohamed M. Hefnawy, Claudiu T. Supuran

**Affiliations:** a Department of Pharmaceutical Chemistry, College of Pharmacy, King Saud University, Riyadh, Saudi Arabia; b Department of Pharmaceutical Organic Chemistry, Faculty of Pharmacy, Cairo, Egypt; c Pharmacognosy and Pharmaceutical Chemistry Department, College of Pharmacy, Taibah University, Al-Madinah Al-Munawarah, Saudi Arabia; d Department of Neurofarba, Sezione di Scienze Farmaceutiche e Nutraceutiche, Università degli Studi di Firenze, Florence, Italy

**Keywords:** Metalloenzyme, quinazolinone, sulphonamide, inhibition, selectivity, molecular docking study

## Abstract

Inhibitory action of newly synthesised 4-(2-(2-substituted-thio-4-oxoquinazolin-3(4H)-yl)ethyl)benzenesulfonamides compounds **2–13** against human carbonic anhydrase (CA, EC 4.2.1.1) (hCA) isoforms I, II, IX, and XII, was evaluated. hCA I was efficiently inhibited by compounds **2–13** with inhibition constants (K_I_s) ranging from 57.8–740.2 nM. Compounds **2**, **3**, **4**, and **12** showed inhibitory action against hCA II with K_I_s between 6.4 and 14.2 nM. CA IX exhibited significant sensitivity to inhibition by derivatives **2–13** with K_I_ values ranging from 7.1 to 93.6 nM. Compounds **2**, **3**, **4**, **8**, **9**, and **12** also exerted potent inhibitory action against hCA XII (K_I_s ranging from 3.1 to 20.2 nM). Molecular docking studies for the most potent compounds **2** and **3** were conducted to exhibit the binding mode towards hCA isoforms as a promising step for SAR analyses which showed similar interaction with co-crystallized ligands. As such, a subset of these mercaptoquinazolin-4(3H)-one compounds represented interesting leads for developing new efficient and selective carbonic anhydrase inhibitors (CAIs) for the management of a variety of diseases including glaucoma, epilepsy, arthritis and cancer.

## Graphical Abstract

**Figure:**
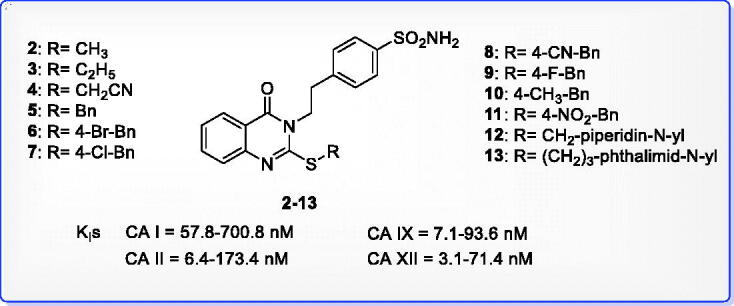

## 1. Introduction

**Figure 1.**
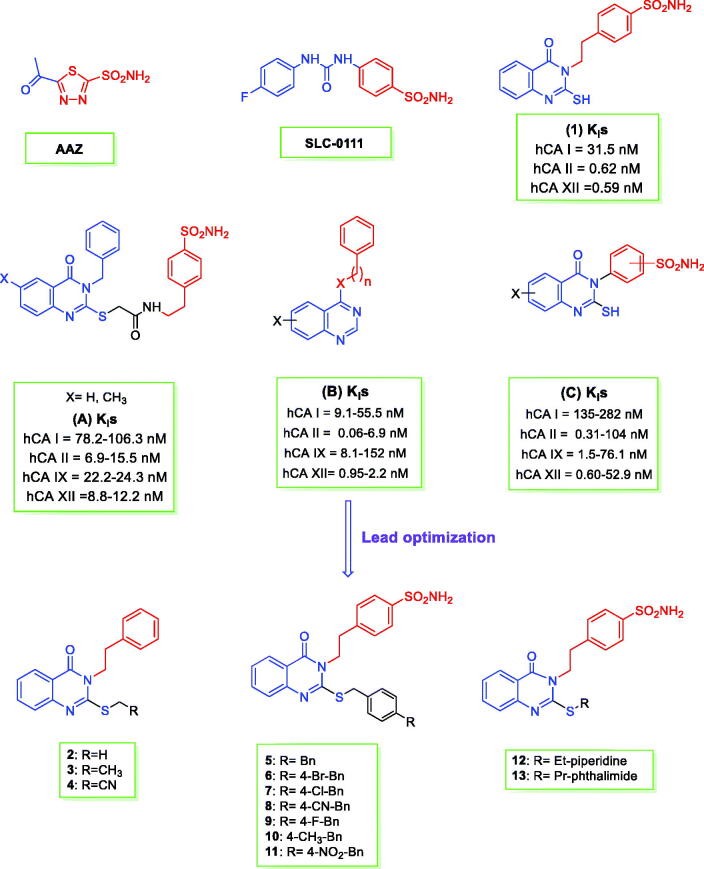
Structures of AAZ, SLC-0111, (**A–C**), and the herein designed quinazoline derivatives (**2–13**) as CAIs.

Carbonic anhydrases (CAs; EC 4.2.1.1) constitute the superfamily of metalloenzymes catalysing the CO_2_ hydration/dehydration reaction. CAs are classified into eight genetically distinct families, named α-, β-, γ-, δ-, ζ-, η-, ɵ-, and ι-CAs^1^^,^^2^. 15 α-class isoenzymes have been detected in humans (h) and are sorted into four different subsets depending on their subcellular localisation: CA I, II, III, VII, VIII, X, XI, XIII are cytosolic proteins, CA VA and VB are present in the mitochondrial matrix, CA VI is a secreted enzyme, CA IV is a glycosylphosphatidylinositol (GPI)-anchored protein and CA IX, XII, and XIV are trans-membrane isoforms^1–3^. hCAs are spread in the human body and are implicated in a plethora of essential physiological processes. As a result, critical pathological conditions might occur upon their dysregulated expression and/or abnormal activity^2^. CA II is the most physiologically relevant isoform and is implicated in disorders such as cerebral oedema, glaucoma (such as CA XII), and epilepsy. It is conversely off-target like CA I, when targeting tumours where CA IX and XII are overexpressed and represent validated targets to combat the growth of both primary tumours and metastasis^4^. Significant similarity exists amongst the active site’s architecture of hCAs making it difficult to produce inhibitors that exhibit net isoform-specificity in action and do not induce side effects as a consequence of CA inhibition^1^. Aromatic sulphonamides constitute the main subset of CAIs developed to date and have been clinically used for decades as diuretic, antiglaucoma, antiobesity, and antiepilepsy medications. The ureidobenzenesulfonamide SLC-0111 (Figure 1), a selective CA IX/XII inhibitor, is currently in Phase II/b clinical trials for the therapy of solid, metastatic tumours^2–6^. A wealth of sulphonamide derivatives have been reported as CAIs^5^^,^^7–19^, COX-2 inhibitors, or antitumor^13^^,^^15^^,^^20–22^. The quinazolinone scaffold is also widely used in medicinal chemistry^23–39^, such as COX-1/2 inhibitors^23^^,^^24^ and antitumor^25–29^^,^^37–39^. 4–(2-(4-Oxo-2-thioxo-1,4-dihydroquinazolin-3(2H)-yl)ethyl)benzenesulfonamide (**1**, Figure 1) showed effective inhibitory activity against a subset of hCA isoforms with subnanomolar inhibition constants^40^. Likewise, a series of 2-((3-benzyl-4-oxo-3,4-dihydroquinazolin-2-yl)thio)-N-(4-sulfamoylphenethyl)amides (**A**, Figure 1) showed nanomolar inhibitory action against a panel of hCAs^39^. Various quinazolin-4-yl-aminobenzenesulfonamide, quinazolin-4-yl-oxy-benzenesulfonamide derivatives (**B**, Figure 1) and 3-(6-iodo-4-oxo-2-thioxo-1,4-dihydroquinazolin-3(2H)-yl)benzenesulfonamide, 3-(2-mercapto-7-fluoro-4(3H)quinazolinon-3-yl)-benzenesulfonamide (**C**, Figure 1) were reported to exert potent inhibitory effect against CA I, II, IX and XII^41^^,^^42^. As observed from SAR analysis of the reported **C** derivatives (Figure 1), it is thought to add an ethyl linker between sulphonamide part and quinazoline scaffold and alkylation of free SH group to increase the lipophilicity as activity parameter in CA enzyme. Therefore, we report the synthesis of a new series of 4-(2-(2-(substituted-thio)-4-oxoquinazolin-3(4H)-yl)ethyl)benzenesulfonamide compounds (**2–13**, Figure 1) and evaluated their inhibitory action against four pharmacologically relevant hCA isoforms, I, II, IX, and XII.

## 2. Materials and methods

### 2.1. Chemistry

Melting points were recorded on a Barnstead 9100 electrothermal melting point apparatus (UK). IR spectra (KBr) were recorded on a FT-IR Perkin-Elmer spectrometer (Perkin Elmer Inc., Waltham, MA). Nuclear magnetic resonance (^1^H and ^13^C NMR) spectra were recorded with Bruker 500 or 700 MHz spectrometers (Zurich, Switzerland) using DMSO-d_6_ as the solvent. Micro-analytical data (C, H, and N) were obtained using a Perkin-Elmer 240 analyser (Perkin Elmer Inc., MA) and agreed with the proposed structures within ±0.4% of the theoretical values. Mass spectra were recorded on a Varian TQ 320 GC/MS/MS mass spectrometer (Varian, Palo Alto, CA).

4-(2-(4-Oxo-2-thioxo-1,4-dihydroquinazolin-3(2*H*)-yl)ethyl)benzenesulfonamide compound **1** was prepared by heating anthranilic acid with 4-(2-isothiocyanatoethyl)benzenesulfonamide in ethanol in the presence of triethylamine^40^.

#### 2.1.1. *General procedure for synthesis of* 4-(2-(2-(substituted-thio)-4-oxoquinazolin-3(4H)-yl)ethyl)benzenesulfonamides *(2–13)*

A mixture of 4-(2-(4-oxo-2-thioxo-1,4-dihydroquinazolin-3(2H)-yl)ethyl)benzenesulfonamide (**1**) (1 mmol, 361 mg), appropriate halide (1 mmol) and potassium carbonate (3 mmol, 415 mg) in 7 ml acetone were stirred at room temperature for 8–13 h. The reaction mixture was filtered and the prepared solid was washed with water and dried.

##### 2.1.1.1. 4-(2-(2-(Methylthio)-4-oxoquinazolin-3(4H)-yl)ethyl)benzenesulfonamide (2)

M.P. 255–257 °C, 91% yield; IR (KBr, cm^−1^) *ν*: 3285, 3237 (NH), 1655 (C=O), 1345, 1159 (O=S=O); ^1^H NMR (700 MHz, DMSO-d_6_): *δ* 8.09 (d, 1H, *J* = 7.78 Hz), 7.89 (d, 3H, *J* = 8.17 Hz), 7.57 (d, 1H, *J* = 8.14), 7.48 (dd, 3H, *J* = 8.24 & 7.70 Hz), 7.35 (s, 2H), 4.27 (t, 2H, *J* = 15.99 Hz), 3.07 (t, 2H, *J* = 16.20 Hz), 2.65 (s, 3H); ^13^C NMR (176 MHz, DMSO-d_6_): *δ* 160.89, 157.38, 147.29, 143.07, 142.41, 135.21, 129.65, 126.89, 126.48, 126.43, 126.39, 119.16, 45.42, 33.58, 15.11; MS; *m*/*z* (375).

##### 2.1.1.2. 4-(2-(2-(Ethylthio)-4-oxoquinazolin-3(4H)-yl)ethyl)benzenesulfonamide (3)

M.P. 193–195 °C, 94% yield; ^1^H NMR (700 MHz, DMSO-d_6_): *δ* 8.09 (d, 1H, *J* = 7.89 Hz), 7.80 (d, 3H, *J* = 8.12 Hz), 7.55 (d, 1H, *J* = 8.12 Hz), 7.47 (d, 3H, *J* = 8.19 Hz), 7.34 (s, 2H), 4.25 (t, 2H, *J* = 15.96 Hz), 3.27 (q, 2H, *J* = 7.30 Hz), 3.06 (t, 2H, *J* = 15.96 Hz), 1.36 (d, 3H, *J* = 7.32 Hz); ^13^C NMR (176 MHz, DMSO-d_6_): *δ* 160.97, 156.68, 147.33, 143.07, 142.41, 135.22, 129.66, 126.87, 126.46, 126.42, 126.38, 119.22, 45.38, 33.58, 26.49, 14.47; MS: *m*/*z* 389.

##### 2.1.1.3. 4-(2-(2-((Cyanomethyl)thio)-4-oxoquinazolin-3(4H)-yl)ethyl)benzenesulfonamide (4)

M.P. 169–170 °C, 90% yield; IR (KBr, cm^−1^) *ν*: 3287, 3232 (NH), 2193 (CN), 1700 (C=O), 1333, 1155 (O=S=O); ^1^H NMR (700 MHz, DMSO-d_6_): *δ* 8.13 (d, 1H, *J* = 7.86 Hz), 7.86 (t, 1H, *J* = 7.61 Hz), 7.80 (d, 2H, *J* = 7.78 Hz), 7.63 (d, 1H, *J* = 8.11 Hz), 7.53 (t, 1H, *J* = 7.51 Hz), 7.49 (d, 2H, *J* = 7.82 Hz), 7.35 (s, 2H), 4.42 (s, 2H), 4.24 (t, 2H, *J* = 15.93 Hz), 3.08 (t, 2H, *J* = 15.95 Hz); ^13^C NMR (176 MHz, DMSO-d_6_): *δ* 160.76, 154.07, 146.88, 143.14, 142.19, 135.48, 129.69, 127.11, 127.01, 126.62, 126.50, 119.41, 117.91, 45.75, 33.62, 18.20; MS: *m*/*z* 400.

##### 2.1.1.4. 4-(2-(2-(Benzylthio)-4-oxoquinazolin-3(4H)-yl)ethyl)benzenesulfonamide (5)

M.P. 251–252 °C, 95% yield; IR (KBr, cm^−1^) *ν*: 3289, 3233 (NH), 1686 (C=O), 1331, 1158 (O=S=O); ^1^H NMR (500 MHz, DMSO-d_6_): *δ* 9.09 (d, 1H, *J* = 7.40 Hz), 7.28 (s, 1H), 7.78 (d, 2H, *J* = 6.80 Hz), 7.64 (d, 1H, *J* = 7.50 Hz), 7.53 (d, 2H, *J* = 6.10 Hz), 7.48 (d, 1H, *J* = 7.45 Hz), 7.44 (d, 2H, *J* = 6.75 Hz), 7.35 (s, 4H), 7.28 (d, 1H, *J* = 6.55 Hz), 4.57 (s, 2H), 4.25 (s, 2H), 3.04 (s, 2H); ^13^C NMR (125 MHz, DMSO-d_6_): *δ* 160.93, 156.26, 147.18, 143.06, 142.33, 137.12, 135.28, 129.88, 129.65, 128.95, 127.90, 126.90, 126.52, 126.45, 45.41, 36.06, 33.61; MS: 451.

##### 2.1.1.5. 4-(2-(2-((4-Bromobenzyl)thio)-4-oxoquinazolin-3(4H)-yl)ethyl)benzenesulfonamide (6)

M.P. 225–227 °C, 92% yield; IR (KBr, cm^−1^) *ν*: 3291, 3226 (NH), 1645 (C=O), 1340, 1155 (O=S=O); ^1^H NMR (500 MHz, DMSO-d_6_): *δ* 8.08 (d, 1H, *J* = 7.26 Hz), 7.78 (t, 3H, *J* = 10. 05 & 7.10 Hz), 7.63 (d, 1H, *J* = 7.37 Hz), 7.48 (dd, 7H, *J* = 11.70 & 19.80 Hz), 7.36 (s, 2H), 4.52 (s, 2H), 4.23 (s, 2H), 3.04 (s, 2H); ^13^C NMR (125 MHz, DMSO-d_6_): *δ* 160.91, 156.02, 147.11, 143.07, 142.34, 137.00, 135.28, 132.07, 131.74, 129.67, 126.90, 126.56, 126.46, 120.97, 119.27, 45.48, 35.21, 33.61; MS: *m*/*z* 530 and 532.

##### 2.1.1.6. 4-(2-(2-((4-Chlorobenzyl)thio)-4-oxoquinazolin-3(4H)-yl)ethyl)benzenesulfonamide (7)

M.P. 244–245 °C, 95% yield; IR (KBr, cm^−1^) *ν*: 3282, 3224 (NH), 1655 (C=O), 1333, 1155 (O=S=O); ^1^H NMR (500 MHz, DMSO-d_6_): *δ* 8.04 (s, 1H), 7.78 (d, 3H, *J* = 6.51 Hz), 7.63 (s, 1H), 7.54 (s, 2H), 7.45 (s, 3H), 7.38 (s, 2H), 7.35 (s, 2H), 4.54 (s, 2H), 4.23 (s, 2H), 3.04 (s, 2H); ^13^C NMR (125 MHz, DMSO-d_6_): *δ* 162.53, 156.04, 147.12, 142.36, 136.56, 135.30, 132.44, 131.71, 129.68, 128.81, 126.89, 126.58, 126.45, 119.26, 116.12, 45.47, 35.15, 33.60; MS; *m*/*z* 486 and 487.

##### 2.1.1.7. 4-(2-(2-((4-Cyanobenzyl)thio)-4-oxoquinazolin-3(4H)-yl)ethyl)benzenesulfonamide (8)

M.P. 265–267 °C, 95% yield; IR (KBr, cm^−1^) *ν*: 3280, 3222 (NH), 2189 (CN), 1654 (C=O), 1334, 1155 (O=S=O); ^1^H NMR (700 MHz, DMSO-d_6_): *δ* 8.08 (dd, 1H, *J* = 7.91 & 1.19 Hz), 7.82 (dd, 1H, *J* = 7.00 & 1.40 Hz), 7.79 (t, 4H, *J* = 17.70 Hz), 7.73 (d, 2H, *J* = 8.28 Hz), 7.62 (d, 1H, *J* = 8.19 Hz), 7.48 (d, 1H, *J* = 7.28 Hz), 7.45 (d, 2H, *J* = 8.19 Hz), 7.37 (s, 2H), 4.62 (s, 2H), 4.24 (t, 2H, *J* = 15.90 Hz), 3.05 (t, 2H, *J* = 15.87 Hz); ^13^C NMR (176 MHz, CDCl_3_/DMSO-d_6_): *δ* 160.90, 155.79, 147.05, 143.83, 143.07, 142.35, 135.31, 132.70, 130.81, 129.70, 126.91, 126.63, 126.46, 126.41, 119.28, 119.23, 110.45, 45.58, 35.31, 33.60; MS: *m*/*z* 476.

##### 2.1.1.8. 4-(2-(2-((4-Fluorobenzyl)thio)-4-oxoquinazolin-3(4H)-yl)ethyl)benzenesulfonamide (9)

M.P. 267–269 °C, 93% yield; IR (KBr, cm^−1^) *ν*: 3285, 3220 (NH), 1663 (C=O), 1336, 1157 (O=S=O); ^1^H NMR (500 MHz, DMSO-d_6_): *δ* 8.08 (d, 1H, *J* = 7.32 Hz), 7.78 (t, 3H, *J* = 12.70 & 6.69 Hz), 7.64 (d, 1H, *J* = 7.68 Hz), 7.57 (s, 2H), 7.44 (t, 3H, *J* = 9.65 & 6.56 Hz), 7.35 (s, 2H), 7.16 (d, 2H, *J* = 8.25 Hz), 4.55 (s, 2H), 4.23 (s, 2H), 3.04 (s, 2H);^13^C NMR (125 MHz, DMSO-d_6_): *δ* 160.92, 156.16, 147.15, 143.06, 142.35, 135.28, 133.55, 131.90, 131.83, 129.66, 126.90, 126.54, 126.45, 119.28, 115.76, 115.59, 45.44, 35.16, 33.60; MS: *m*/*z* 469.

##### 2.1.1.9. 4-(2-(2-((4-Methylbenzyl)thio)-4-oxoquinazolin-3(4H)-yl)ethyl)benzenesulfonamide (10)

M.P. 227–228 °C, 92% yield; IR (KBr, cm^−1^) *ν*: 3287, 3221 (NH), 1656 (C=O), 1334, 1156 (O=S=O); ^1^H NMR (700 MHz, DMSO-d_6_): *δ* 8.09 (d, 1H, *J* = 7.84 Hz), 7.81 (d, 1H, *J* = 6.79 Hz), 7.78 (d, 2H, *J* = 7.98 Hz), 7.64 (d, 1H, *J* = 8.12 Hz), 7.47 (t, 1H, *J* = 8.11 Hz), 7.44 (d, 2H, *J* = 7.91 Hz), 7.40 (d, 2H, *J* = 7.70 Hz), 7.35 (s, 2H), 7.14 (d, 2H, *J* = 7.70 Hz), 4.52 (s, 2H), 4.23 (t, 2H, *J* = 15.89 Hz), 3.03 (t, 2H, *J* = 15.92 Hz), 2.27 (s, 3H); ^13^C NMR (176 MHz, DMSO-d_6_): *δ* 160.91, 156.02, 147.11, 143.07, 142.34, 137.00, 135.28, 132.07, 131.74, 129.67, 126.90, 126.56, 126.46, 120.97, 119.27, 45.48, 35.21, 33.61; MS: *m*/*z* 465.

##### 2.1.1.10. 4-(2-(2-((4-Nitrobenzyl)thio)-4-oxoquinazolin-3(4H)-yl)ethyl)benzenesulfonamide (11)

M.P. 210–211 °C, 90% yield; IR (KBr, cm^−1^) *ν*: 3279, 3226 (NH), 1661 (C=O), 1326, 1161 (O=S=O); ^1^H NMR (500 MHz, DMSO-d_6_): *δ* 8.19 (d, 2H, *J* = 6.56 Hz), 8.08 (d, 1H, *J* = 6.10 Hz), 7.80 (t, 5H, *J* = 8.30 & 8.26 Hz), 7.64b (d, 1H, *J* = 5.55 Hz), 7.46 (s, 3H), 7.36 (s, 2H), 4.68 (s, 2H), 4.25 (s, 2H), 3.05 (s, 2H); ^13^C NMR (125 MHz, DMSO-d_6_): *δ* 160.90, 155.71, 147.06, 146.06, 143.07, 142.36, 135.31, 131.08, 129.71, 126.89, 126.65, 126.45, 123.86, 119.27, 45.60, 35.02, 33.60; MS: *m*/*z* 496.

##### 2.1.1.11. 4-(2-(4-Oxo-2-((2-(piperidin-1-yl)ethyl)thio)quinazolin-3(4H)-yl)ethyl)benzenesulfonamide (12)

M.P. 198–199 °C, 92% yield; ^1^H NMR (700 MHz, DMSO-d_6_): *δ* 7.08 (dd, 1H, *J* = 9.18 & 6.64 Hz), 7.79 (d, 3H, *J* = 8.22 Hz), 7.53 (d, 1H, *J* = 8.05 Hz), 7.47 (dd, 3H, *J* = 8.26 & 6.70 Hz), 7.36 (s, 2H), 4.26 (t, 2H, *J* = 16.04 Hz), 3.41 (t, 2H, *J* = 14.25 Hz), 3.07 (t, 2H, *J* = 16.01 Hz), 2.65 (s, 2H), 2.51 (t, 2H, *J* = 3.55 Hz), 2.48 (d, 2H, *J* = 6.54 Hz), 1.50 (d, 4H, *J* = 4.35 Hz), 1.38 (s, 2H); ^13^C NMR (176 MHz, DMSO-d_6_): *δ* 160.95, 156.81, 147.27, 143.08, 142.43, 135.24, 129.65, 126.88, 126.46, 126.38, 126.34, 119.19, 57.48, 54.15, 45.38, 33.59, 31.17, 29.37, 25.93, 24.36; MS: *m*/*z* 472.

##### 2.1.1.12. 4-(2-(2-((3-(1,3-Dioxoisoindolin-2-yl)propyl)thio)-4-oxoquinazolin-3(4H)-yl)ethyl)benzenesulfonamide (13)

M.P. 205–207 °C, 91% yield; IR (KBr, cm^−1^) *ν*: 3277, 3228 (NH), 1701 (C=O), 1328, 1159 (O=S=O); ^1^H NMR (700 MHz, DMSO-d_6_): *δ* 8.05 (d, 1H, *J* = 7.84 Hz), 7.88–7.83 (m, 4H), 7.79 (d, 2H, *J* = 7.91 Hz), 7.68 (p, 1H, *J* = 8.15, 14.14 Hz), 7.54 (d, 2H, *J* = 8.26 Hz), 7.42 (p, 1H, *J* = 17.15 & 7.03 Hz), 7.36 (s, 2H), 7.20 (d, 1H, *J* = 8.12 Hz), 4.21 (t, 2H, *J* = 15.86 Hz), 3.75 (t, 2H, *J* = 6.49 Hz), 3.31 (t, 2H, *J* = 7.18 Hz), 3.03 (t, 2H, *J* = 16.07 Hz), 2.10–2.06 (m, 2H); ^13^C NMR (176 MHz, DMSO-d_6_): *δ* 168.52, 160.88, 156.36, 147.11, 143.05, 142.37, 135.05, 134.87, 132.14, 129.62, 127.74, 126.84, 126.47, 126.36, 126.14, 123.51, 119.17, 45.28, 37.00, 33.54, 29.22, 28.21; MS: *m*/*z* 547.

### 2.2. CA inhibition

The hCA I, II, IX, and XII isoenzyme inhibition assay was performed according to the reported method using SX.18 MV-R stopped-flow instrument (Applied Photophysics, Oxford, UK)^43–45^. All CA isoforms were recombinant isoforms obtained in-house, as reported earlier^46^^,^^47^.

### 2.3. Molecular docking method

Molecular docking was carried out according to the previously reported methods^24^^,^^28^^,^^29^^,^^37–39^^,^^48–52^ using MOE 2008.10 from the Chemical Computing Group Inc^53^.

## 3. Results and discussion

### 3.1. Chemistry

**Scheme 1.**
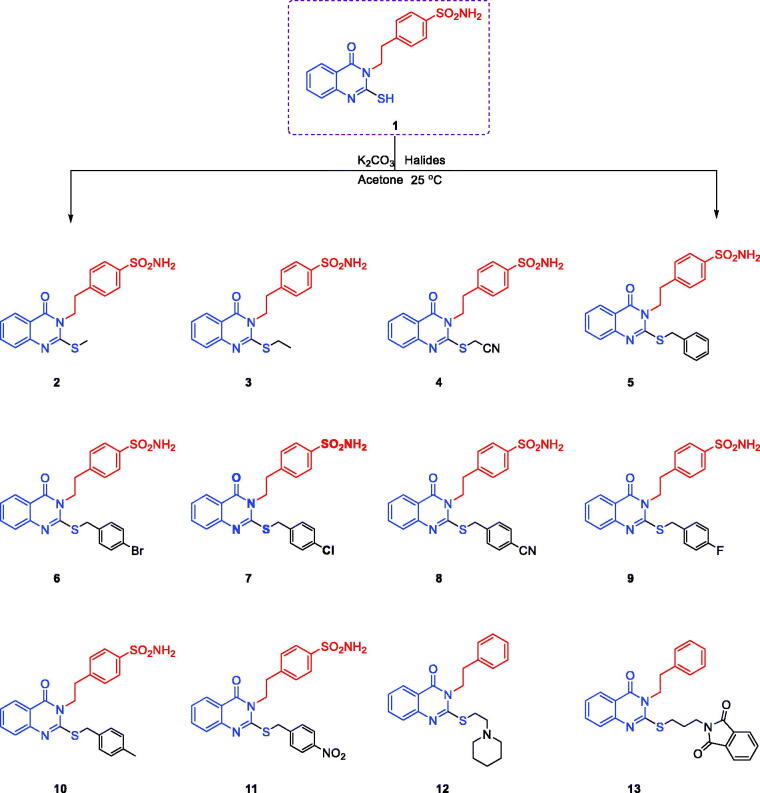
Synthesis of 4–(2–(2-(substituted-thio)-4-oxoquinazolin-3(4H)-yl)ethyl)benzenesulfonamides **2–13.**

4-(2-(4-Oxo-2-thioxo-1,4-dihydroquinazolin-3(2H)-yl)ethyl)benzenesulfonamide (**1**) was obtained through the reaction between anthranilic acid, 4-(2-isothiocyanatoethyl)benzenesulfonamide and triethylamine in ethanol^40^^,^^54^ (Scheme 1). Its yield was 93%. Stirring of compound **1** with potassium carbonate in acetone and different alkyl-halides or aralkyl-halides produced the corresponding 4-(2-(2-(substituted-thio)-4-oxoquinazolin-3(4H)-yl)ethyl)benzenesulfonamides **2–13** with 90–95% yield. Various spectral analyses were performed to confirm the structures of compounds **2–13**. The formation of target compounds was assessed by the disappearance of thioamide proton (NH–C = S) at 13.03 ppm in ^1^H NMR and thione moiety (NH–C = S) at 175.29 ppm in the ^13^C NMR spectra, together with presence of the new thio-substituted moieties (S–R), that were confirmed by ^1^H NMR and ^13^C NMR spectra.

### 3.2. CA inhibitory activity

**Figure 2.**
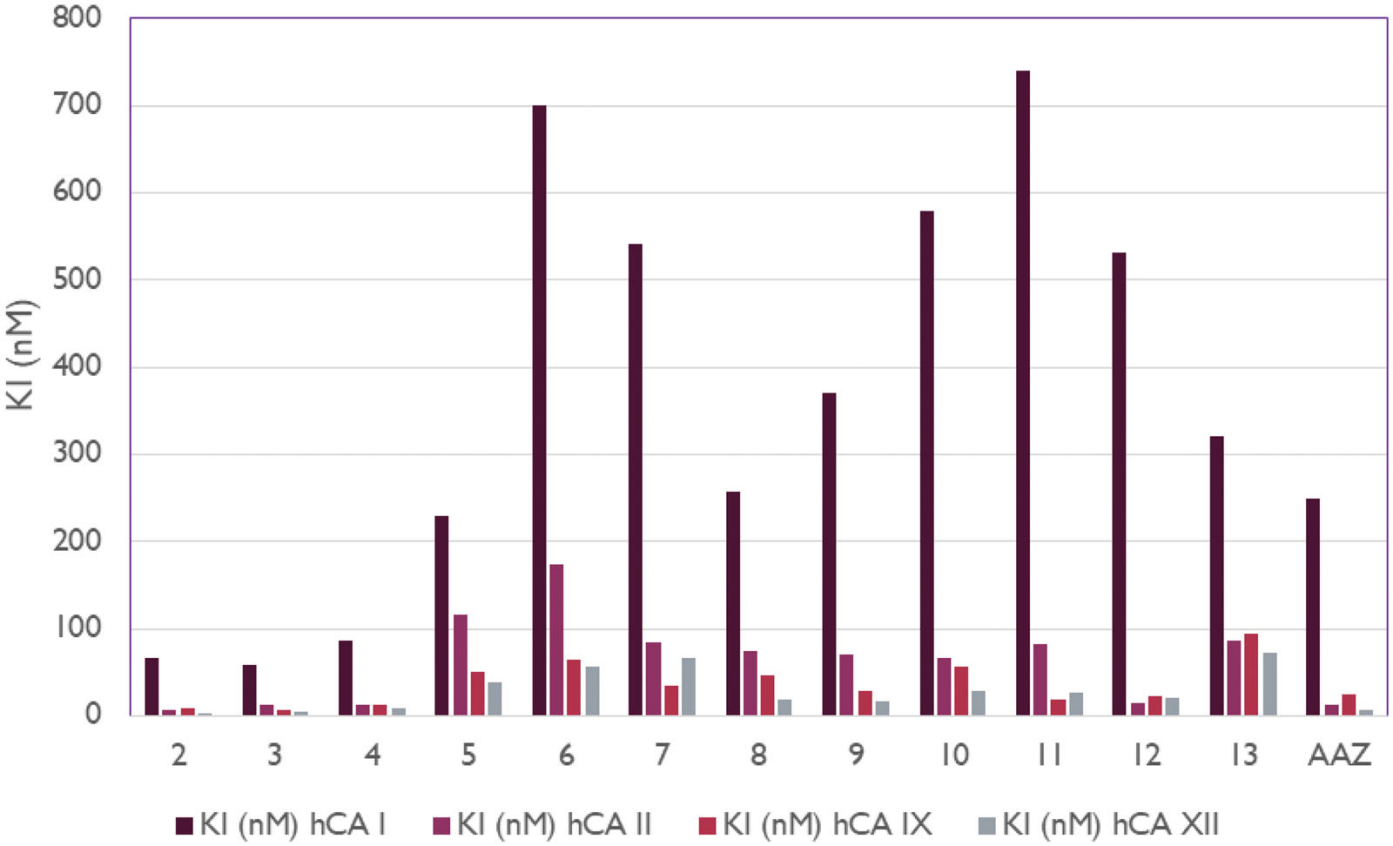
Carbonic anhydrase inhibition of 2-substituted-mercapto-4(3H|)-quinazolinone derivatives **2–13**.

The CAI activity of newly produced 4-(2-(2-(substituted-thio)-4-oxoquinazolin-3(4H)-yl)ethyl)benzenesulfonamides **2**-**13** against hCA I, II, IV, and IX isoforms was measured by a Stopped-Flow kinetic assay^48^ and compared to acetazolamide (AAZ), a standard sulphonamide inhibitor (Table 1, Figure 2). The hCA I was effectively inhibited by compounds **2–13** with inhibition constant (K_I_) values in the range of 57.8–740.2 nM, (AAZ: K_I_, 250.0 nM). Compounds **2**, **3**, **4**, and **12** showed to be potent hCA II inhibitors, with K_I_ values between 6.4 and 14.2 nM, which were greater than or nearly identical to that of AAZ (K_I_, 12.0 nM). Compounds **7–11** and **13** showed modest hCA II inhibitory activity with K_I_s ranging between 66.5 and 86.6 nM, whereas compounds **5** and **6** showed a weak inhibitory activity with K_I_s values of 115.3 and 173.4 nM, respectively. Compounds **2–13** displayed potent hCA IX inhibitory activity with K_I_ values ranging from 7.1 to 93.6 nM (AAZ K_I_, 25.0 nM). Quinazoline derivatives **2**, **3**, **4**, **8**, **9**, and **12** possessed potent hCA XII inhibitory activities with K_I_ values ranging between 3.1–20.2 nM (AAZ K_I_, 5.7 nM). On the other hand, compounds **5**, **10**, and **11** exerted moderate hCA XII inhibitory activities with K_I_ values between 25.6–38.4 nM, whereas compounds **6**, **7**, and **13** had weak hCA XII inhibitory activities with K_I_ values in the range of 57.6–71.4 nM (Table 1, Figure 2).

**Table 1.** Inhibition constant values of 2-ethylquinazoline derivatives **2–13** and standard sulphonamide inhibitor acetazolamide (AAZ) against human CA isoforms hCA I, II, IX, and XII as determined by a stopped flow, CO_2_ hydrase assay^48^.
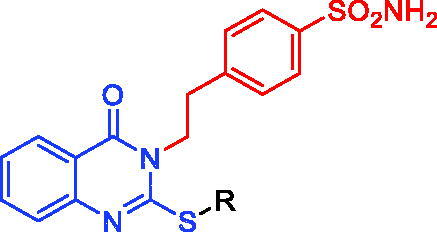

Cmpound	*R*	*K*_I_ (nM)^a^			
		hCA I	hCA II	hCA IX	hCA XII
**1**	H	31.5	0.62		0.59
**2**	CH_3_	67.0	6.4	9.5	3.1
**3**	C_2_H_5_	57.8	11.6	7.1	3.9
**4**	CH_2_CN	85.5	13.5	12.6	8.6
**5**	Bn	229.4	115.3	50.7	38.4
**6**	4-Br-Bn	700.8	173.4	64.1	57.0
**7**	4-Cl-Bn	541.9	84.2	34.2	65.6
**8**	4-CN-Bn	256.8	74.1	47.3	17.6
**9**	4-F-Bn	370.1	69.3	28.9	17.2
**10**	4-CH_3_-Bn	578.7	66.5	57.1	28.2
**11**	4-NO_2_-Bn	740.2	81.7	19.3	25.6
**12**	CH_2_CH_2_-piperidin-N-yl	532.2	14.2	22.4	20.2
**13**	(CH_2_)_3_-phthalimid-N-yl	320.5	86.6	93.6	71.4
AAZ		250.0	12.0	25.0	5.7

^a^Mean from 3 different assays, by a stopped flow technique (errors were in the range of ±5 to 10% of the reported values).

The following structure-activity relationship (SAR) can be drawn on the basis of the inhibition data shown in Table 1.

- SAR analysis for hCA I inhibition indicated that: (1) 2-(aliphatic-thio)quinazolin-4(3H)-one like compounds **2–4** (K_I_ values between 57.8 and 85.5 nM) were more active than the corresponding 2-(benzylthio)quinazolin-4(3H)-ones (K_I_ values between 229.4 and 740.2 nM) **5–11**, 4–(2-(4-oxo-2-((2-(piperidin-1-yl)ethyl)thio)quinazolin-3(4H)-yl)ethyl)benzenesulfonamide (**12**) (K_I_, 532.2 nM) and 4–(2-(2-((3-(1,3-dioxoisoindolin-2-yl)propyl)thio)-4-oxoquinazolin-3(4H)-yl)ethyl)benzenesulfonamide (**13**) (K_I_, 320.5 nM); (2) 2-(benzylthio)quinazolin-4(3H)-one (**5**) (K_I_, 229.4 nM) was more potent than the corresponding 4**-**substituted-2-(benzylthio)quinazolin-4(3H)-ones such as compounds **6–11** and **13** (K_I_ values ranging from 256.8 to 740.2 nM); (3) 4-(2-(2-((4-cyanobenzyl)thio)-4-oxoquinazolin-3(4H)-yl)ethyl)benzenesulfonamide (**8**) (K_I_, 256.8 nM) was more effective than other 2-((4-substituted-benzyl)thio)quinazolin-4(3H)-ones **6**, **7**, **9**, **10** and **11** (K_I_ values between 370.1 and 740.2 nM); (4) 4-(2-(2-(ethylthio)-4-oxoquinazolin-3(4H)-yl)ethyl)benzenesulfonamide (**2**) with K_I_ of 57.8 nM was more potent than 4-(2-(4-oxo-2-((2-(piperidin-1-yl)ethyl)thio)quinazolin-3(4H)-yl)ethyl)benzenesulfonamide (**12**) with K_I_ of 532.2 nM; (5) hCA I inhibitory activity (K_I_, 85.5 nM) of 4-(2-(2-((cyanomethyl)thio)-4-oxoquinazolin-3(4H)-yl)ethyl)benzenesulfonamide (**4**) was more potent than 4-(2-(2-((4-cyanobenzyl)thio)-4-oxoquinazolin-3(4H)-yl)ethyl)benzenesulfonamide (**8**) with K_I_ of 256.8 nM; (6) substitution of benzyl group of compound **5** (K_I_ of 229.4 nM) by propylphthalimide moiety produced compound **13** with significant decrease of CA I activity (K_I_, 320.5 nM).
- SAR analysis for hCA II inhibition indicated that: (1) 2-(aliphatic-thio)quinazolin-4(3H)-one compounds such as compounds **2**, **3**, **4** and **12** (K_I_ values ranging from 6.4 to 14.2 nM) were more active than the corresponding 2-(benzylthio)quinazolin-4(3H)-ones **5–11** and 4-(2-(2-((3-(1,3-dioxoisoindolin-2-yl)propyl)thio)-4-oxoquinazolin-3(4H)-yl)ethyl)benzenesulfonamide (**13**) with K_I_ values between 66.5 and 173.4 nM; (2) the introduction of electron withdrawing groups such as 4-Cl, 4-CN, 4-F, 4-NO_2_ or electron donating group such as 4-CH_3_ at the benzyl moiety of compound **5** (K_I,_ 115.3 nM) produced compounds **7–11** with moderate increase in the CA I activity (K_I_ values ranging between 66.5 and 84.2 nM); (3) 4-(2-(2-(ethylthio)-4-oxoquinazolin-3(4H)-yl)ethyl)benzenesulfonamide (**2**) with K_I_, 11.6 nM was more potent than 4-(2-(4-oxo-2-((2-(piperidin-1-yl)ethyl)thio)quinazolin-3(4H)-yl)ethyl)benzenesulfonamide (**12**) with K_I_, 14.2 nM; (4) For hCA I, inhibitory activity (K_I_, 13.5 nM) of 4-(2-(2-((cyanomethyl)thio)-4-oxoquinazolin-3(4H)-yl)ethyl)benzenesulfonamide (**4**) was more potent than 4-(2-(2-((4-cyanobenzyl)thio)-4-oxoquinazolin-3(4H)-yl)ethyl)benzenesulfonamide (**8**) with K_I_, 74.1 nM; (5) substitution of benzyl group in compound **5** (K_I_, 115.3 nM) by propylphthalimide moiety produced compound **13** with significant increase in the CA activity (K_I,_ 86.6 nM).
- SAR analysis for hCA IX inhibition indicated that: (1) 2-(aliphatic-thio)quinazolin-4(3H)-one such as compounds **2–4** (K_I_ values ranging between 7.1 and 12.6 nM) were more active than the corresponding 2-(benzylthio)quinazolin-4(3H)-one **5–11**, 4-(2-(4-oxo-2-((2-(piperidin-1-yl)ethyl)thio)quinazolin-3(4H)-yl)ethyl)benzenesulfonamide (**12**) and 4-(2-(2-((3-(1,3-dioxoisoindolin-2-yl)propyl)thio)-4-oxoquinazolin-3(4H)-yl)ethyl)benzenesulfonamide (**13**) (K_I_ values in the range of 19.3–93.6 nM); (2) the introduction of electron withdrawing groups such as 4-Cl, 4-F, or 4-NO_2_ at the benzyl moiety in compound **5** (K_I_, 50.7 nM) produced compounds **7**, **9**, and **11** with significant increase in the CA I activity (K_I_ values ranging between 19.3 and 34.2 nM); (3) 4-(2-(2-(ethylthio)-4-oxoquinazolin-3(4H)-yl)ethyl)benzenesulfonamide (**2**) with K_I_ of 7.1 nM was more potent than 4-(2-(4-oxo-2-((2-(piperidin-1-yl)ethyl)thio)quinazolin-3(4H)-yl)ethyl)benzenesulfonamide (**12**) with K_I_ value of 22.2 nM; (4) hCA I inhibitory activity of 4-(2-(2-((cyanomethyl)thio)-4-oxoquinazolin-3(4H)-yl)ethyl)benzenesulfonamide (**4**) (K_I_, 12.6 nM) was more potent than 4-(2-(2-((4-cyanobenzyl)thio)-4-oxoquinazolin-3(4H)-yl)ethyl)benzenesulfonamide (**8**) with K_I_ value of 47.3 nM; (5) substitution of benzyl group in compound **5** (K_I_, 50.7 nM) by propylphthalimide moiety produced compound **13** with significant increase in the CA activity (K_I_, 93.6 nM).
- SAR analysis for hCA XII inhibition indicated that: (1) 2-(aliphatic-thio)quinazolin-4(3H)-one compounds such as compounds **2**-**4** (K_I_ values between 3.1 and 8.6 nM) were more active than the corresponding 2-(benzylthio)quinazolin-4(3H)-one **5–11**, 4-(2-(4-oxo-2-((2-(piperidin-1-yl)ethyl)thio)quinazolin-3(4H)-yl)ethyl)benzenesulfonamide (**12**) and 4-(2-(2-((3-(1,3-dioxoisoindolin-2-yl)propyl)thio)-4-oxoquinazolin-3(4H)-yl)ethyl)benzenesulfonamide (**13**) (K_I_ values ranging from 17.2 to 71.4 nM); (2) the introduction of electron withdrawing groups such as 4-CN, 4-F, 4-NO_2_ or electron donating group such as 4-CH_3_ at the benzyl moiety in compound **5** (K_I_, 115.3 nM) produced compounds **8–11** which significantly increased the CAI activity (K_I_, 17.2–28.2 nM), while introduction of 4-Br and 4-Cl groups at the benzyl moiety in compound **5** gave compounds **6** and **7** which significantly increased the CAI activity (K_I_ values, 57.0 and 65.6 nM, respectively); 3) 4-(2-(2-(ethylthio)-4-oxoquinazolin-3(4H)-yl)ethyl)benzenesulfonamide (**2**) with K_I_ of 3.9 nM was more potent than 4-(2-(4-oxo-2-((2-(piperidin-1-yl)ethyl)thio)quinazolin-3(4H)-yl)ethyl)benzenesulfonamide (**12**) with K_I_ of 20.2 nM; (4) hCA XII inhibitory activity of 4-(2-(2-((cyanomethyl)thio)-4-oxoquinazolin-3(4H)-yl)ethyl)benzenesulfonamide (**4**) (K_I_, 8.6 nM) was more potent than 4-(2-(2-((4-cyanobenzyl)thio)-4-oxoquinazolin-3(4H)-yl)ethyl)benzenesulfonamide (**8**) with K_I_ of 14.6 nM; (5) substitution of benzyl group in compound **5** (K_I_ of 38.4 nM) by propylphthalimide moiety produced compound **13** with a significant decrease in CAI activity (K_I_, 71.4 nM).

### 3.3. Molecular docking studies

The docking simulations between the hCAs targets, and the most active compounds such as **2** and **3**, as well as least active compound such as **6** compared with the prototype **1** lead compound were performed using MOE Suite^53^.

#### 3.3.1. Docking of compounds 2, 3, and 6 with hCA isoenzymes

**Figure 3.**
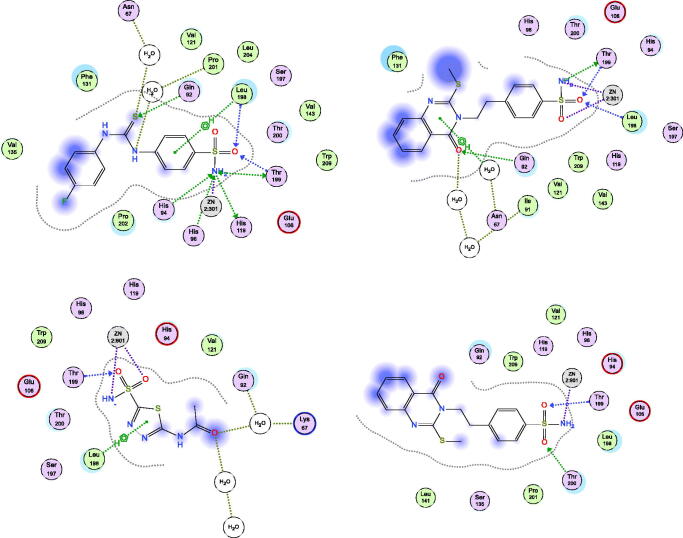
Docking modes of compound **2** in the binding pockets of CA isoenzymes II and XII. Interactions between the protein (PDB IDs: 5ULN and 1JD0). Predicted binding modes of co-crystallized inhibitor (upper left panel) and compound **2** (upper right panel) with hCA-II target as well as co-crystallized inhibitor (lower left panel) and compound **2** (lower right panel) with hCA-XII target.

**Figure 4.**
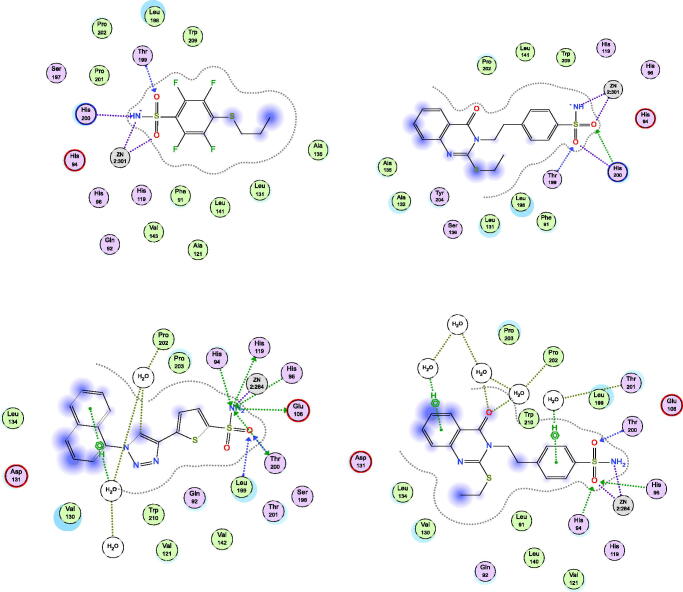
Docking modes of compound **3** in the binding pockets of CA isoenzymes I and IX. Interactions between the protein (PDB IDs: 4WR7, 5FL4). Predicted binding modes of co-crystallized inhibitor (upper left panel) and compound **3** (upper right panel) with hCA-I target as well as co-crystallized inhibitor (lower left panel) and compound **3** (lower right panel) with hCA-IX target.

**Figure 5.**
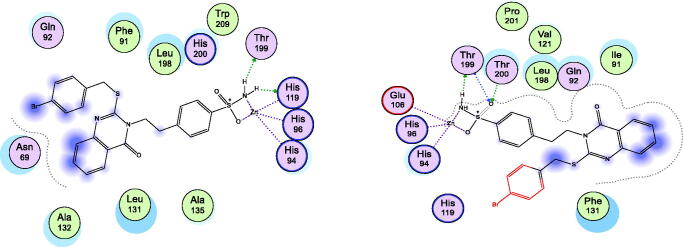
Docking modes of compound **6** as the least active example in the binding pockets of CA isoenzymes I and II. Interactions between the protein (PDB IDs: 4WR7, 5ULN). Predicted binding modes of compound **6** with CA-I; right panel, and CA-II; left panel.

The most active methyl derivative **2** was docked with the binding pockets of the hCA isoforms II and XII, utilising the different protein crystal structures; 5ULN and 1JD0 downloaded from Protein Data Bank store^55^^,^^56^. As shown in Figure 3, and Table 2, the results suggested that the compound **2** displayed similar patterns to the co-crystallized ligands. Firstly, the docking pose of compound **2** in the complex with isoform II formed bidentate chelate with SO_2_NH_2_ fragment with zinc metal at a distance range of 2.45–2.01 Å. The sulphonyl part was stabilised by three strong hydrogen bonds through the residues, Thr199 and Leu198 at average bond distance of 2.35–2.22 Å. Moreover, the carbonyl moiety of 4(3H)quinazolinone was stabilised by direct and indirect hydrogen bonding between water and polar residues Gln92 and Asn67 at distance of 2.25 Å as co-crystallized inhibitor S-atom does. In addition, hydrophobic interactions were experienced with methylthioether fragment through Phe131 residue. Secondly, the docking poses of compound **2** in the complex with isoform XII exhibited interactions like the co-crystallized inhibitor especially the SO_2_NH_2_ part with Zinc metal and stabilising Thr199 and Thr200 residues. In addition, hydrophobic interactions were experienced with methylthioether fragment through Leu141 residue. The compound **3** was docked with the binding pockets in the hCA isoforms I and IX, utilising the different protein crystal structures; 4WR7 and 5FL4 downloaded from Protein Data Bank store^57^^,^^58^. As shown in Figure 4 and Table 2, the results suggested that the compound **3** interacted with both active sites in a similar fashion to the co-crystallized ligands in the pockets. Firstly, the docking pose of compound **3** in the complex with isoform I, consisted of a long, narrow tunnel, leading to a cavity that contained the catalytic Zn^2+^ ion chelated with SO_2_NH_2_ fragment (2.45–2.01 Å) and a sulphonyl part stabilised by two strong hydrogen bonds through the residues, Thr199 and His200 (2.38-2.41 Å). Moreover, different hydrophobic aromatic interactions were also formed with ethylthioether, phenethyl, and 4(3H)quinazolinone moieties through pockets of Leu131, Leu198, Pro202, Leu141, Trp209, Ala135, and Ala132 residues. Secondly, the docking pose of compound **3** in the complex with isoform IX, revealed that the cavity contained the catalytic Zn^2+^ ion forming bidentate chelate with SO_2_NH_2_ fragment (2.32-2.12 Å) and the sulphonyl part was stabilised by three hydrogen bonds through the residues, Thr200, His96 and His94 (2.44-1.99 Å). Different hydrophobic aromatic interactions were formed with ethylthioether, phenethyl, and 4(3H)quinazolinone moieties through pockets of Val130, Leu134, and Leu91 residues. The 4(3H)quinazolinone was modulated through molecules of H_2_O in the pocket by polar interactions. In addition, the aryl moiety of benzene sulphonamide formed a H_2_O-mediated π-π interactions with certain aromatic amino acids. Thr201 might also play an important role in increasing their binding affinity for the enzyme. In addition, the lease active compound **6** was placed in the hCA I binding cavity (Figure 5, right panel) and results showed that certain factors affecting the incorrect placement like the insertion of S-bromophenyl ring among polar Gln92 and Asn69 residues and disorienting of planer quinazolinone to Leu131 residue. Moreover, the docking of the least active compound **6** into the hCA-II pocket (Figure 5, left panel) revealed the intolerance of S side chain bromophenyl moiety into the His119 polar part leading to protrusion out of the pocket and so appeared incompatible with pocket residue that makes it low active.

**Table 2.** Description of the docking data of selected target compounds **2** and **3**.

Compound	Target	Fragments	Residues (distance, Å)	Interactions	Binding energy (dG, kcal/mol)
**1**	hCA-II	SO_2_NH_2_	Thr199, 2.31 His119, 2.72 His94, 2.45	Hydrogen bonding	−24.11
			Zn metal, 2.42	Coordination bonding	
		Phenethyl	Trp209	Hydrophobic	
		4(3H)-Quinazolinone	Gln92, Lys67 (H_2_O)	Hydrogen bonding	
	hCA-XII	SO_2_NH_2_	Thr199, 2.22 His119, 2.81 His94, 2.46 His96, 2.25	Hydrogen bonding	−23.65
		Phenethyl	Leu198	Hydrophobic	
		4(3H)-Quinazolinone	Gln92 (H_2_O), Lys67 (H_2_O)	Hydrogen bonding	
**2**	hCA-II	SO_2_NH_2_	Thr199, 2.31 Thr199, 2.42 Leu198, 2.46	Hydrogen bonding	−24.9
			Zn metal, 2.51	Coordination bonding	
		Phenethyl	His96	Aromatic stacking	
		4(3H)-Quinazolinone	Asn67 (H_2_O)	Aromatic stacking	
			Gln92, 2.44 Ile91 (H^2^O)	Hydrogen bonding	
			Phe131	Hydrophobic	
		Methylthioether	Phe131	Hydrophobic	
	hCA-XII	SO_2_NH_2_	Thr199, 2.50 Thr200, 2.47	Hydrogen bonding	−27.5
			Zn metal, 2.38	Coordination bonding	
		Phenethyl	Pro201, Ser128	Hydrophobic	
		4(3H)-Quinazolinone	Gln92, 2.44	Hydrogen bonding	
		Methylthioether	Leu141	Hydrophobic	
**3**	hCA-I	SO_2_NH_2_	His200, 2.57 Thr199, 2.45	Hydrogen bonding	−19.5
			Zn metal, 2.54	Coordination bonding	
		Phenethyl	Leu141, Trp202	Hydrophobic	
		4(3H)-Quinazolinone	Ala135, Ala132, Tyr204	Hydrophobic	
		Ethylthioether	Leu198, Leu131, Tyr204	Hydrophobic	
	hCA-IX	SO_2_NH_2_	Thr200, 2.47 His94, 2.36 His96, 2.48	Hydrogen bonding	−25.6
			Zn metal, 2.44	Coordination bonding	
		Phenethyl	Thr201 (H_2_O)	Aromatic stacking	
			Trp210	Hydrophobic	
		4(3H)-Quinazolinone	Pro202 (H_2_O)	Hydrogen bonding	
			Asp131, Leu134	Hydrophobic	
		Ethylthioether	Val130, Leu134	Hydrophobic	

^a^ The data reported in the table were extracted from MOE programme showing the corresponding amino acid residues in enzyme pocket, corresponding fragment of ligands, interaction distances, types of interaction, and their binding energy to prototype 1 and selected active compounds.

**Figure 6.**
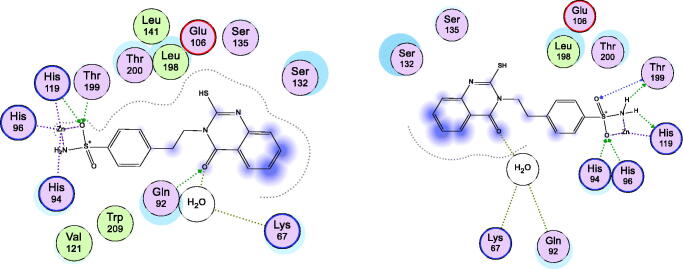
Docking modes of the lead compound **1** in the binding pockets of CA isoenzymes II (Right) and XII (Left) with (PDB IDs: 5ULN and 1JD0) respectively.

However, the lead compound **1** was docked into the pockets of hCA II and XII (Figure 6, Table 2) for comparing its behaviour that showed the loss of SH role in the interactions compared to the potent active **2** and **3** derivatives. These overall docking findings proved that the S-alkylated derivatives exhibited good binding interactions better than the lead compound **1**.

## 4. Conclusions

A new series of 4-(2-(2-substituted-thio-4-oxoquinazolin-3(4H)-yl)ethyl)benzenesulfonamide derivatives **2–13** were synthesised and assessed *in vitro* for CA inhibition in comparison to AAZ as reference drug. 4–(2-(2-Aliphatic-thio-4-oxoquinazolin-3(4H)-yl)ethyl)benzenesulfonamide derivatives **2–4** showed efficient inhibitory activity against hCA I, hCA II, h IX and hCA XII with K_I_ values ranging between 57.8–85.5, 6.4–13.5, 7.1–12.6, and 3.1–8.6 nM, respectively, which were better or had the same activity as AAZ as standard drug with K_I_ values of 250, 12.0, 25.0, and 5.7 nM, respectively. 4–(2-(2-Aliphatic-thio-4-oxoquinazolin-3(4H)-yl)ethyl)benzenesulfonamide derivatives **2–4** were more active than the corresponding 2-(benzylthio)quinazolin-4(3H)-ones **5–11**, 4–(2-(4-oxo-2-((2-(piperidin-1-yl)ethyl)thio)quinazolin-3(4H)-yl)ethyl)benzenesulfonamide (**12**) and 4-(2-(2-((3-(1,3-dioxoisoindolin-2-yl)propyl)thio)-4-oxoquinazolin-3(4H)-yl)ethyl)benzenesulfonamide (**13**) but lower than 4-(2-(4-Oxo-2-thioxo-1,4-dihydroquinazolin-3(2H)-yl)ethyl)benzenesulfonamide (**1**) as parent compound. Molecular docking studies for compounds **2** and **3** were done and exhibited specific binding modes for hCA isoforms as comparable interaction with lead compound **1**.

## References

[1] Alterio V, Fiore AD, D’Ambrosio K, et al. Multiple binding modes of inhibitors to carbonic anhydrases: how to design specific drugs targeting 15 different isoforms? Chem Rev 2012;112:4421–68. 22607219 10.1021/cr200176r

[2] Supuran CT. Carbonic anhydrases. Bioorg Med Chem 2013;21:1377–8. 23452985 10.1016/j.bmc.2013.02.026

[3] Borras J, Scozzafava A, Menabuoni L, et al. Carbonic anhydrase inhibitors: synthesis of water-soluble, topically effective intraocular pressure lowering aromatic/heterocyclic sulfonamides containing 8-quinoline-sulfonyl moieties: is the tail more important than the ring? Bioorg Med Chem 1999;7:2397–406. 10632049 10.1016/s0968-0896(99)00190-x

[4] Scozzafava A, Menabuoni L, Mincione F, et al. Carbonic anhydrase inhibitors: synthesis of sulfonamides incorporating dtpa tails and of their zinc complexes with powerful topical antiglaucoma properties. Bioorg Med Chem Lett 2001;11:575–82. 11229775 10.1016/s0960-894x(00)00722-8

[5] Scozzafava A, Menabuoni L, Mincione F, et al. Synthesis of water-soluble, topically effective, intraocular pressure-lowering aromatic/heterocyclic sulfonamides containing cationic or anionic moieties: is the tail more important than the ring? J Med Chem 1999;42:2641–50. 10411484 10.1021/jm9900523

[6] Sheldrick GM. A short history of SHELX. Acta Crystallographica. Section A, Foundations Crystallography 2008;64:112–22. 10.1107/S0108767307043930 18156677

[7] Abdel-Aziz AA, El-Azab AS, Abu El-Enin MA, et al. Synthesis of novel isoindoline-1,3-dione-based oximes and benzenesulfonamide hydrazones as selective inhibitors of the tumor-associated carbonic anhydrase IX. Bioorg Chem 2018;80:706–13. 30064081 10.1016/j.bioorg.2018.07.027

[8] Abdel-Aziz AA, El-Azab AS, Ekinci D, et al. Investigation of arenesulfonyl-2-imidazolidinones as potent carbonic anhydrase inhibitors. J Enz Inhibition Med Chem 2015;30:81–4. 10.3109/14756366.2014.880696 24666299

[9] Angeli A, Abdel-Aziz AA, Nocentini A, et al. Synthesis and carbonic anhydrase inhibition of polycyclic imides incorporating N-benzenesulfonamide moieties. Bioorg Med Chem 2017;25:5373–9. 28789908 10.1016/j.bmc.2017.07.056

[10] Mohamed MA, Abdel-Aziz AA, Sakr HM, et al. Synthesis and human/bacterial carbonic anhydrase inhibition with a series of sulfonamides incorporating phthalimido moieties. Bioorg Med Chem 2017;25:2524–9. 28318894 10.1016/j.bmc.2017.03.017

[11] Abdel-Aziz AA, Angeli A, El-Azab AS, et al. Synthesis and biological evaluation of cyclic imides incorporating benzenesulfonamide moieties as carbonic anhydrase I, II, IV and IX inhibitors. Bioorg Med Chem 2017;25:1666–71. 28161252 10.1016/j.bmc.2017.01.032

[12] Abdel-Aziz AA, El-Azab AS, Ceruso M, Supuran CT. Carbonic anhydrase inhibitory activity of sulfonamides and carboxylic acids incorporating cyclic imide scaffolds. Bioorg Med Chem Lett 2014;24:5185–9. 25442309 10.1016/j.bmcl.2014.09.076

[13] Alaa A-M, El-Azab AS, El-Subbagh HI, et al. Design, synthesis, single-crystal and preliminary antitumor activity of novel arenesulfonylimidazolidin-2-ones. Bioorg Med Chem Lett 2012;22:2008–14. 22318157 10.1016/j.bmcl.2012.01.036

[14] El-Azab AS, Abdel-Aziz AA, Ayyad RR, et al. Inhibition of carbonic anhydrase isoforms I, II, IV, VII and XII with carboxylates and sulfonamides incorporating phthalimide/phthalic anhydride scaffolds. Bioorg Med Chem 2016;24:20–5. 26678172 10.1016/j.bmc.2015.11.034

[15] Abdel-Aziz AA, Angeli A, El-Azab AS, et al. Synthesis and anti-inflammatory activity of sulfonamides and carboxylates incorporating trimellitimides: dual cyclooxygenase/carbonic anhydrase inhibitory actions. Bioorg Chem 2019;84:260–8. 30508771 10.1016/j.bioorg.2018.11.033

[16] Abdel-Aziz AA, El-Azab AS, Ghiaty AH, et al. 4-Substituted benzenesulfonamides featuring cyclic imides moieties exhibit potent and isoform-selective carbonic anhydrase II/IX inhibition. Bioorg Chem 2019;83:198–204. 30380448 10.1016/j.bioorg.2018.10.037

[17] Abdel-Aziz AA, El-Azab AS, Bua S, et al. Design, synthesis, and carbonic anhydrase inhibition activity of benzenesulfonamide-linked novel pyrazoline derivatives. Bioorg Chem 2019;87:425–31. 30921744 10.1016/j.bioorg.2019.03.052

[18] El-Azab AS, Abdel-Aziz AA, Bua S, et al. Synthesis and comparative carbonic anhydrase inhibition of new Schiff’s bases incorporating benzenesulfonamide, methanesulfonamide, and methylsulfonylbenzene scaffolds. Bioorg Chem 2019;92:103225. 31493707 10.1016/j.bioorg.2019.103225

[19] El-Azab AS, Abdel-Aziz AA, Bua S, et al. New anthranilic acid-incorporating N-benzenesulfonamidophthalimides as potent inhibitors of carbonic anhydrases I, II, IX, and XII: synthesis, in vitro testing, and in silico assessment. Eur J Med Chem 2019;181:111573. 31394463 10.1016/j.ejmech.2019.111573

[20] Abdel-Aziz AA, El-Azab AS, Abou-Zeid LA, et al. Synthesis, anti-inflammatory, analgesic and COX-1/2 inhibition activities of anilides based on 5,5-diphenylimidazolidine-2,4-dione scaffold: molecular docking studies. Eur J Med Chem 2016;115:121–31. 26999325 10.1016/j.ejmech.2016.03.011

[21] Abdel-Aziz AA, El-Azab AS, Alanazi AM, et al. Synthesis and potential antitumor activity of 7-(4-substituted piperazin-1-yl)-4-oxoquinolines based on ciprofloxacin and norfloxacin scaffolds: in silico studies. J Enzyme Inhibition Med Chem 2016;31:796–809. 10.3109/14756366.2015.1069288 26226179

[22] Al-Suwaidan IA, Alanazi AM, El-Azab AS, et al. Molecular design, synthesis and biological evaluation of cyclic imides bearing benzenesulfonamide fragment as potential COX-2 inhibitors. Part 2. Bioorg Med Chem Lett 2013;23:2601–5. 23528298 10.1016/j.bmcl.2013.02.107

[23] Abdel-Aziz AA, Abou-Zeid LA, ElTahir KE, et al. Design, synthesis of 2,3-disubstitued 4(3H)-quinazolinone derivatives as anti-inflammatory and analgesic agents: COX-1/2 inhibitory activities and molecular docking studies. Bioorg Med Chem 2016;24:3818–28. 27344214 10.1016/j.bmc.2016.06.026

[24] Abdel-Aziz AA, Abou-Zeid LA, ElTahir KEH, et al. Synthesis, anti-inflammatory, analgesic, COX-1/2 inhibitory activities and molecular docking studies of substituted 2-mercapto-4(3H)-quinazolinones. Eur J Med Chem 2016;121:410–21. 27318118 10.1016/j.ejmech.2016.05.066

[25] Alanazi AM, Abdel-Aziz AA-M, Al-Suwaidan IA, et al. Design, synthesis and biological evaluation of some novel substituted quinazolines as antitumor agents. Eur J Med Chem 2014;79:446–54. 24763265 10.1016/j.ejmech.2014.04.029

[26] Alanazi AM, Al-Suwaidan IA, Abdel-Aziz AA-M, et al. Design, synthesis and biological evaluation of some novel substituted 2-mercapto-3-phenethylquinazolines as antitumor agents. Med Chem Res 2013;22:5566–77.

[27] Al-Obaid AM, Abdel-Hamide SG, El-Kashef HA, et al. Substituted quinazolines, part 3. Synthesis, in vitro antitumor activity and molecular modeling study of certain 2-thieno-4(3H)-quinazolinone analogs. Eur J Med Chem 2009;44:2379–91. 18950904 10.1016/j.ejmech.2008.09.015

[28] Al-Suwaidan IA, Abdel-Aziz AA, Shawer TZ, et al. Synthesis, antitumor activity and molecular docking study of some novel 3-benzyl-4(3H)quinazolinone analogues. J Enzyme Inhibition Med Chem 2016;31:78–89. 10.3109/14756366.2015.1004059 25815668

[29] Al-Suwaidan AM, Alanazi AA, Abdel-Aziz MA, Mohamed AS. Design, synthesis and biological evaluation of 2-mercapto-3-phenethylquinazoline bearing anilide fragments as potential antitumor agents: molecular docking study. Bioorg Med Chem Lett 2013;23:3935–41. 23683592 10.1016/j.bmcl.2013.04.056

[30] El-Azab AS, Abdel-Hamide SG, Sayed-Ahmed MM, et al. Novel 4 (3H)-quinazolinone analogs: synthesis and anticonvulsant activity. Med Chem Res 2013;22:2815–27.

[31] El-Azab AS, Al-Omar MA, Abdel-Aziz AA, et al. Design, synthesis and biological evaluation of novel quinazoline derivatives as potential antitumor agents: molecular docking study. Eur J Med Chem 2010;45:4188–98. 20599299 10.1016/j.ejmech.2010.06.013

[32] El-Azab AS, Eltahir KE. Synthesis and anticonvulsant evaluation of some new 2,3,8-trisubstituted-4(3H)-quinazoline derivatives. Bioorg Med Chem Lett 2012;22:327–33. 22137344 10.1016/j.bmcl.2011.11.007

[33] El-Azab AS, Abdel-Aziz A-M, Ng SW, Tiekink ER. 6-Methyl-3-phenyl-2-sulfanylidene-1, 2, 3, 4-tetrahydroquinazolin-4-one. Acta Crystallographica Section E: Structure Reports Online 2012;68:o862. 10.1107/S1600536812007301 PMC3297914 22412717

[34] El-Azab AS, Alaa A-M, Bua S, et al. Synthesis of benzensulfonamides linked to quinazoline scaffolds as novel carbonic anhydrase inhibitors. Bioorg Chem 2019;87:78–90. 30878812 10.1016/j.bioorg.2019.03.007

[35] El-Azab AS, ElTahir KE, Attia SM. Synthesis and anticonvulsant evaluation of some novel 4 (3H)-quinazolinones. Monatshefte Für Chemie-Chem Month 2011;142:837–48.

[36] Mohamed MA, Ayyad RR, Shawer TZ, et al. Synthesis and antitumor evaluation of trimethoxyanilides based on 4(3H)-quinazolinone scaffolds. Eur J Med Chem 2016;112:106–13. 26890117 10.1016/j.ejmech.2016.02.002

[37] Alanazi AM, Abdel-Aziz AA, Shawer TZ, et al. El-Azab, Synthesis, antitumor and antimicrobial activity of some new 6-methyl-3-phenyl-4(3H)-quinazolinone analogues: in silico studies. J Enzyme Inhibition Med Chem 2016;31:721–35. 10.3109/14756366.2015.1060482 26162029

[38] El-Azab AS, Al-Dhfyan A, Abdel-Aziz AA, et al. Synthesis, anticancer and apoptosis-inducing activities of quinazoline-isatin conjugates: epidermal growth factor receptor-tyrosine kinase assay and molecular docking studies. J Enzyme Inhibition Med Chem 2017;32:935–44. 10.1080/14756366.2017.1344981 PMC6445199 28718672

[39] El-Azab AS, Abdel-Aziz AA, Ghabbour HA, Al-Gendy MA. Synthesis, in vitro antitumour activity, and molecular docking study of novel 2-substituted mercapto-3-(3,4,5-trimethoxybenzyl)-4(3H)-quinazolinone analogues. J Enzyme Inhibition Med Chem 2017;32:1229–39. 10.1080/14756366.2017.1368504 PMC6010141 28948843

[40] Bozdag M, Alafeefy AM, Carta F, et al. Synthesis 4-[2-(2-mercapto-4-oxo-4H-quinazolin-3-yl)-ethyl]-benzenesulfonamides with subnanomolar carbonic anhydrase II and XII inhibitory properties. Bioorg Med Chem 2016;24:4100–7. 27396930 10.1016/j.bmc.2016.06.052

[41] Bozdag M, Alafeefy AM, Vullo D, et al. Benzenesulfonamides incorporating bulky aromatic/heterocyclic tails with potent carbonic anhydrase inhibitory activity. Bioorg Med Chem 2015;23:7751–64. 26639945 10.1016/j.bmc.2015.11.023

[42] Bozdag M, Alafeefy AM, Altamimi AM, et al. Synthesis of new 3-(2-mercapto-4-oxo-4H-quinazolin-3-yl)-benzenesulfonamides with strong inhibition properties against the tumor associated carbonic anhydrases IX and XII. Bioorg Med Chem 2017;25:2782–8. 28389112 10.1016/j.bmc.2017.03.054

[43] Akurathi V, Dubois L, Lieuwes NG, et al. Synthesis and biological evaluation of a 99mTc-labelled sulfonamide conjugate for in vivo visualization of carbonic anhydrase IX expression in tumor hypoxia. Nuclear Med Biol 2010;37:557–64. 10.1016/j.nucmedbio.2010.02.006 20610160

[44] Khalifah RG. The carbon dioxide hydration activity of carbonic anhydrase. I. Stop-flow kinetic studies on the native human isoenzymes B and C. J Biological Chem 1971;246:2561–73. 4994926

[45] Nocentini A, Trallori E, Singh S, et al. 4-Hydroxy-3-nitro-5-ureido-benzenesulfonamides selectively target the tumor-associated carbonic anhydrase isoforms IX and XII showing hypoxia-enhanced antiproliferative profiles. J Med Chem 2018;61:10860–74. 30433782 10.1021/acs.jmedchem.8b01504

[46] Nocentini A, Gratteri P, Supuran CT. Phosphorus versus sulfur: discovery of benzenephosphonamidates as versatile sulfonamide-mimic chemotypes acting as carbonic anhydrase inhibitors. Chem 2019;25:1188–92. 10.1002/chem.201805039 30411821

[47] Nocentini A, Lucidi A, Perut F, et al. Supuran, alpha,gamma-Diketocarboxylic acids and their esters act as carbonic anhydrase IX and XII selective inhibitors. ACS Med Chem Lett 2019;10:661–5. 30996814 10.1021/acsmedchemlett.9b00023 PMC6466837

[48] El-Husseiny WM, El-Sayed MA, Abdel-Aziz NI, et al. Structural alterations based on naproxen scaffold: synthesis, evaluation of antitumor activity and COX-2 inhibition, and molecular docking. Eur J Med Chem 2018;158:134–43. 30216848 10.1016/j.ejmech.2018.09.007

[49] Abdel-Sayed MA, Bayomi SM, El-Sherbeny MA, et al. Synthesis, anti-inflammatory, analgesic, COX-1/2 inhibition activities and molecular docking study of pyrazoline derivatives. Bioorg Med Chem 2016;24:2032–42. 27025563 10.1016/j.bmc.2016.03.032

[50] Alanazi AM, El-Azab AS, Al-Suwaidan IA, et al. Structure-based design of phthalimide derivatives as potential cyclooxygenase-2 (COX-2) inhibitors: anti-inflammatory and analgesic activities. Eur J Med Chem 2015;92:115–23. 25549551 10.1016/j.ejmech.2014.12.039

[51] Al-Suwaidan IA, Abdel-Aziz NI, El-Azab AS, et al. Antitumor evaluation and molecular docking study of substituted 2-benzylidenebutane-1,3-dione, 2-hydrazonobutane-1,3-dione and trifluoromethyl-1H-pyrazole analogues. J Enz Inhib Med Chem 2015;30:679–87. 10.3109/14756366.2014.960863 25472776

[52] El-Sayed MA, Abdel-Aziz NI, Abdel-Aziz AA, et al. Synthesis, biological evaluation and molecular modeling study of pyrazole and pyrazoline derivatives as selective COX-2 inhibitors and anti-inflammatory agents. Part 2. Bioorg Med Chem 2012;20:3306–16. 22516672 10.1016/j.bmc.2012.03.044

[53] MOE (Molecular Operating Environment). Chemical Computing Group M, Quebec, Canada 2012. Available from: http://www.chemcomp.com [last accessed 30 Nov 2019].

[54] Alkahtani HM, Abdalla AN, Obaidullah AJ, et al. Synthesis, cytotoxic evaluation, and molecular docking studies of novel quinazoline derivatives with benzenesulfonamide and anilide tails: dual inhibitors of EGFR/HER2. Bioorg Chem 2020;95:103461. 31838290 10.1016/j.bioorg.2019.103461

[55] Nocentini A, Ceruso M, Bua S, et al. Discovery of beta-adrenergic receptors blocker-carbonic anhydrase inhibitor hybrids for multitargeted antiglaucoma therapy. J Med Chem 2018;61:5380–94. 29851481 10.1021/acs.jmedchem.8b00625

[56] Whittington DA, Waheed A, Ulmasov B, et al. Crystal structure of the dimeric extracellular domain of human carbonic anhydrase XII, a bitopic membrane protein overexpressed in certain cancer tumor cells. Proc Nat Acad Sci 2001;98:9545–50. 11493685 10.1073/pnas.161301298 PMC55489

[57] Zubriene A, Smirnoviene J, Smirnov A, et al. Intrinsic thermodynamics of 4-substituted-2,3,5,6-tetrafluorobenzenesulfonamide binding to carbonic anhydrases by isothermal titration calorimetry. Biophysical Chem 2015;205:51–65. 10.1016/j.bpc.2015.05.009 26079542

[58] Leitans J, Kazaks A, Balode A, et al. Efficient expression and crystallization system of cancer-associated carbonic anhydrase isoform IX. J Med Chem 2015;58:9004–9. 26522624 10.1021/acs.jmedchem.5b01343

